# Supplementary material for: TFE3 regulates whole‐body energy metabolism in cooperation with TFEB
Source: EMBO Mol Med. 2017 Mar 10;9(5):605–21. doi: 10.15252/emmm.201607204 (PMC5412821; doi:10.15252/emmm.201607204)
Supplement: Supplementary file 1 — Appendix [file EMMM-9-605-s001.pdf]

TFE3 REGULATES WHOLE BODY ENERGY METABOLISM IN COOPERATION WITH TFEB

Nunzia Pastore[1,2], Anna Vainshtein[1,2], Tiemo J. Klisch[1,2], Andrea Armani[3], Tuong Huynh[1,2], Niculin J. Herz[1,2], Elena V. Polishchuk[4], Marco Sandri[3,5] and Andrea Ballabio[1,2,4,6]

Appendix Table of Content:

-Appendix Materials and Methods

-Appendix Figures

-Appendix Figure legends

-Appendix Tables

Appendix Material and Methods

AAV vectors production and injection

The human *TFE3* coding sequence was cloned into the pAAV-CMV-EGFP plasmid (Tessitore et al, 2008) by replacing the enhanced green fluorescence protein (EGFP) sequence. The AAV vectors were produced and characterized by the Telethon Institute of Genetics and Medicine (TIGEM, Naples, Italy) AAV Vector Core. pAAV2.1-CMV-hTFE3 and pAAV2.1-CMV-EGFP were triple-transfected in subconfluent 293 cells along with pAd-Helper and pack2/1 packaging plasmids as described previously (Xiao et al, 1999). Recombinant vectors were purified by two rounds of CsCl gradient centrifugation, as described previously (Xiao et al, 1999). Vector titers, expressed as genome copies per milliliter (GC/ml), were assessed by both PCR quantification and dot-blot analysis. Mice were intramuscularly (i.m.) injected with a total dose of 10^{11} GC of AAV2.1 vector preparation. Muscles were collected 8 weeks after injection and frozen in liquid nitrogen for subsequent analysis.

Protein Carbonyls detection

Carbonylation of liver proteins was detected by using the OxyBlot Protein Oxidation Detection Kit from Millipore following the manufacturer's instructions.

Appendix Figure S1

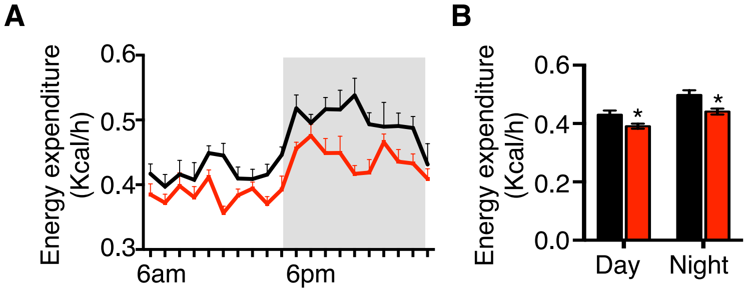

Appendix Figure S1. TFE3 depletion alters energy expenditure

A Energy expenditure (EE) in WT (black line) (***n***=5) and *Tfe3* KO mice (red line) (***n***=4). Grey areas indicate dark periods (6 PM to 6 AM). Data are presented as mean ± SEM.

B Bar graph represents average EE values during day and night (***n***=5 per group). Data are presented as mean ± SEM. Student's two-tailed *t*-test: Day *$\boldsymbol{P}$=0.05; Night *$\boldsymbol{P}$=0.017.

Appendix Figure S2

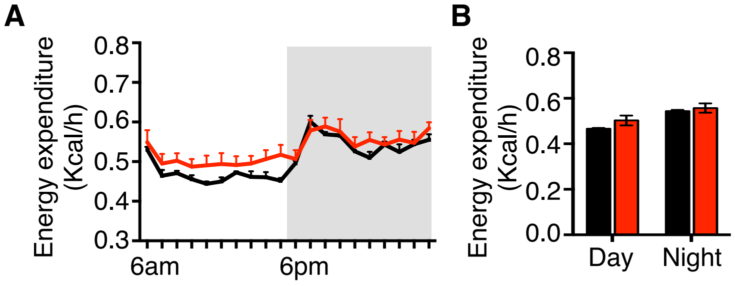

Appendix Figure S2. Energy expenditure

A Energy expenditure (EE) in WT (black line) and *Tfe3* KO mice (red line) after 1 month of HFD (***n***=5 per group). Grey areas indicate dark periods (6 PM to 6 AM). Data are presented as mean ± SEM.

B Bar graph represents average EE values during day and night (***n***=5 per group). Data are presented as mean ± SEM.

Appendix Figure S3

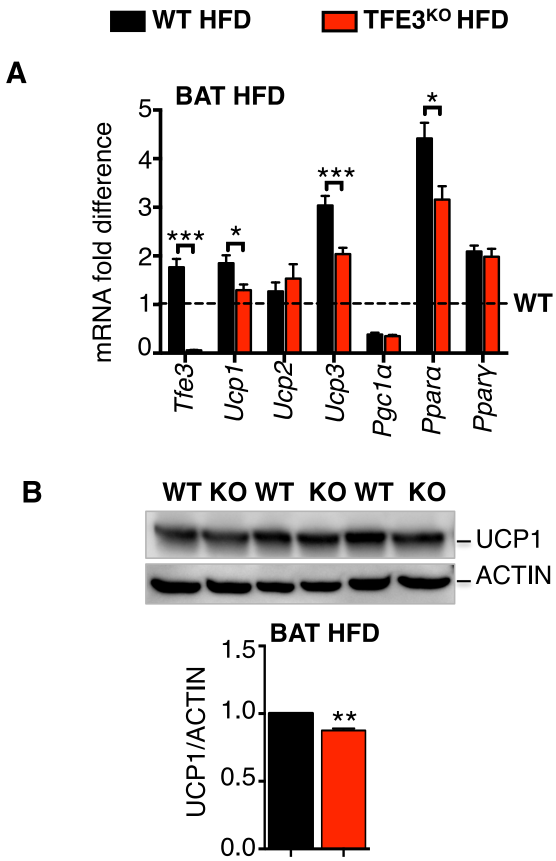

Appendix Figure S3. TFE3 regulates thermogenesis genes in BAT

A Uncoupling protein (*Ucp*) and β-oxidation gene expression in brown adipose tissues (BAT) from WT and *Tfe3* KO mice fed a HFD for 18 weeks (***n***=10 per group). Dashed line represents WT mice fed a chow diet. Data are presented as mean ± SEM. Student's two-tailed *t*-test: *Tfe3* ****P*<0.0001; *Ucp1* **P*=0.0213; *Ucp3* ****P*=0.0006; *Pparα* **P*=0.0114.

B UCP1 immunoblot and relative quantification from WT and *Tfe3* KO mice fed a HFD for 18 weeks. Data are presented as mean ± SEM. Student's two-tailed *t*-test: ***P*=0.0011.

Appendix Figure S4

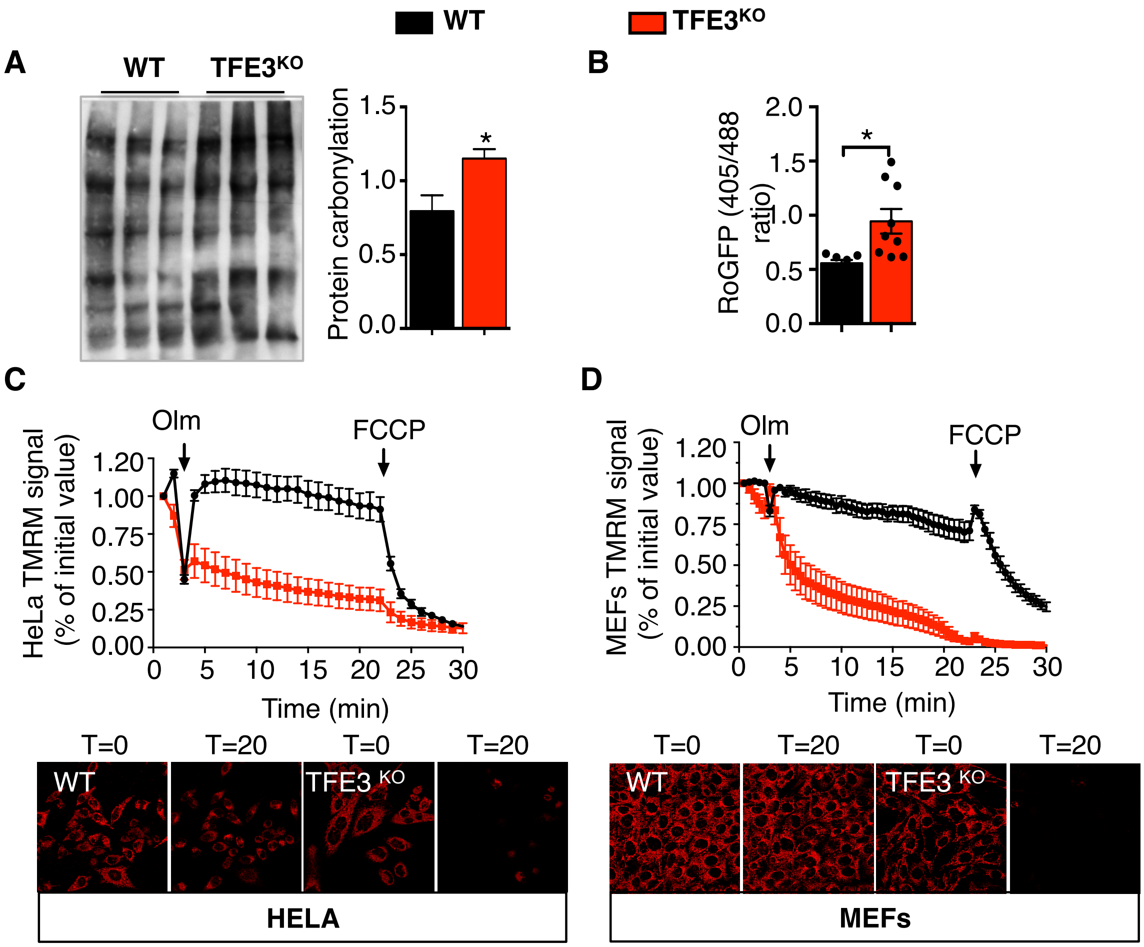

Appendix Figure S4. TFE3 regulates mitochondrial function

A Overall protein carbonylation of WT and *Tfe3* KO livers revealed by Oxyblot. A representative immunoblot for carbonylated proteins is depicted on the left, and densitometric quantification of the carbonylated proteins is represented by the graph on the right. Data are presented as mean ± SEM. Student's two-tailed *t*-test: *Tfe3* ***P*=0.0462.

B Quantification of the RoGFP fluorescence intensity 405/488 emission ratio. ROS production was monitored by transfection of the ROS sensor Matrix-roGFP plasmid in WT (***n***=7) and *Tfe3* KO (***n***=9) MEFs. Data are presented as mean ± SEM. Student's two-tailed *t*-test: ****P***=0.0110.

C-D Mitochondrial membrane potential of WT (***n***=4) and *Tfe3* KO (***n***=7) HeLa (C), and WT (***n***=5) and *Tfe3* KO (***n***=8) MEFs (D) and representative images of TMRM signal. Where indicated, oligomycin (olm) or protonophore carbonylcyanide-p-trifluoromethoxyphenyl hydrazone (FCCP) were added. Data are presented as mean ± SEM.

Appendix Table S1. Metabolic parameters of 2-months-old WT and *Tfe3* KO mice 24h after fasting. ALT, AST, CPK and LDH values are reported in U/L along with the relative *p* value calculated by Student's two-tailed *t*-test.

		**ALT**	**AST**	**CPK**	**LDH**
**Fed**	WT	45±13.4	67.75±14.6	273±110.7	280±92.7
	KO	21.75±1.8	60.5±9.6	418±122.6	253.75±51.8
	p value	0.138	0.69	0.44	0.81
**Fasted**	WT	25.5±4.3	55.5±11.06	203.25±59.8	176.75±32.1
	KO	86.5±49.4	154±85.6	1693.3±958.17	402.25±85.19
	p value	0.05	0.067	0.024	0.048

Appendix Table S2. Gene ontology analysis of the 136 mitochondria-related genes identified as TFE3 targets (Betschinger et al, 2013) (*p* 2,38*E-23).

**Biological Processes in which the TFE3_Mitochondrial Targets are enriched:**	**N GENES**	**SYMBOLS**
GO:0000266~mitochondrial fission	3	FIS1, BAX, MUL1
GO:0000302~response to reactive oxygen species	6	GPX4, BCL2, TXNRD2, PRDX5, SOD2, GLRX2
GO:0001666~response to hypoxia	4	LONP1, BCL2, ABAT, SOD2
GO:0001836~release of cytochrome c from mitochondria	6	BID, BAK1, BBC3, BCL2, BAX, SOD2
GO:0006006~glucose metabolic process	5	PDK1, PDK4, PCK2, OGDH, CPT1A
GO:0006066~alcohol metabolic process	6	PDK1, CPT1B, PDK4, PCK2, OGDH, CPT1A
GO:0006084~acetyl-CoA metabolic process	6	NNT, ACO2, SUCLG1, CS, IDH2, SUCLA2
GO:0006119~oxidative phosphorylation	11	ATP5D, NDUFS4, NDUFB6, NDUFV1, NDUFS8, ATP5F1, ATP5O, ATP5G1, ATP5A1, ATP5G3, UQCRB
GO:0006120~mitochondrial electron transport, NADH to ubiquinone	4	NDUFS4, NDUFB6, NDUFV1, NDUFS8
GO:0006139~nucleobase, nucleoside, nucleotide and nucleic acid metabolic process	23	ATP5D, PHB, CYCS, AK3, ATP5F1, ESR2, ATP5G1, ATP5G3, SOD2, GLRX2, MTHFD1, LONP1, SLC25A13, BAX, MARS2, TFB2M, ATP5O, ATP5A1, POLG2, FPGS, DNAJA3, LRPPRC, DUT
GO:0006200~ATP catabolic process	4	ATP5D, LONP1, ATP5O, ATP5A1
GO:0006626~protein targeting to mitochondrion	9	BID, GRPEL1, TIMM17A, TIMM9, TIMM10, TIMM13, MIPEP, TIMM44, TOMM34
GO:0006635~fatty acid beta-oxidation	6	CPT1B, ACADS, ECHS1, BDH2, DECR1, CPT1A
GO:0006732~coenzyme metabolic process	14	ACO2, SUCLG1, CS, NFS1, COQ7, SOD2, GLRX2, COQ4, MTHFD1, NNT, GPX4, IDH2, FPGS, SUCLA2
GO:0006754~ATP biosynthetic process	8	ATP5D, SLC25A13, ATP5F1, AK3, ATP5O, ATP5G1, ATP5A1, ATP5G3
GO:0006800~oxygen and reactive oxygen species metabolic process	6	NDUFS4, GPX4, BCL2, NDUFA13, MPV17, SOD2

GO:0006811~ion transport	16	ATP5D, CPT1B, ATP5F1, ATP5G1, VDAC2, ATP5G3, VDAC1, SLC25A12, NNT, SLC25A13, PSEN1, SLC25A22, ATP5O, SLC25A1, ATP5A1, SCO2
GO:0006818~hydrogen transport	7	ATP5D, NNT, ATP5F1, ATP5O, ATP5G1, ATP5A1, ATP5G3
GO:0006820~anion transport	7	SLC25A12, SLC25A13, PSEN1, SLC25A22, SLC25A1, VDAC2, VDAC1
GO:0006839~mitochondrial transport	21	ATP5D, BID, CPT1B, GRPEL1, TIMM17A, TIMM10, TIMM13, MIPEP, TIMM44, SLC25A12, BAK1, SLC25A13, PSEN1, BAX, BCL2, TIMM9, SLC25A27, ATP5O, SLC25A1, SLC25A15, TOMM34
GO:0006865~amino acid transport	7	SLC25A12, CPT1B, CLN3, SLC25A13, PSEN1, SLC25A22, SLC25A15
GO:0006874~cellular calcium ion homeostasis	5	BAK1, CLN3, PSEN1, BCL2, BAX
GO:0006886~intracellular protein transport	12	BID, GRPEL1, TIMM17A, TIMM9, TRAK1, TIMM10, NDUFA13, TIMM13, MIPEP, TIMM44, HSPA9, TOMM34
GO:0006979~response to oxidative stress	9	LONP1, PSEN1, GPX4, BCL2, NDUFS8, TXNRD2, PRDX5, SOD2, GLRX2
GO:0007005~mitochondrion organization	24	BID, SPG7, GRPEL1, MSTO1, TIMM17A, TIMM10, MPV17, MUL1, TIMM13, MIPEP, TIMM44, SOD2, BAK1, FIS1, LONP1, NDUFS4, BBC3, BAX, BCL2, TIMM9, NDUFS8, TFB2M, DNAJA3, TOMM34
GO:0007006~mitochondrial membrane organization	7	BID, BAK1, BCL2, BAX, TIMM9, TIMM10, TIMM13
GO:0007007~inner mitochondrial membrane organization	3	TIMM9, TIMM10, TIMM13
GO:0007584~response to nutrient	3	BCKDHA, BCL2, BCKDHB
GO:0008635~activation of caspase activity by cytochrome c	3	BAK1, BAX, CYCS
GO:0008637~apoptotic mitochondrial changes	6	BID, BAK1, BBC3, BCL2, BAX, SOD2
GO:0009060~aerobic respiration	8	NNT, ACO2, SUCLG1, CS, IDH2, SUCLA2, SURF1, UQCRB
GO:0010035~response to inorganic substance	11	SLC25A12, ATP5D, D2HGDH, SLC25A13, GPX4, BCL2, ABAT, TXNRD2, PRDX5, SOD2, GLRX2
GO:0010522~regulation of calcium ion transport into cytosol	3	BAK1, BCL2, BAX
GO:0015711~organic anion transport	5	SLC25A12, SLC25A13, PSEN1, SLC25A22, SLC25A1
GO:0015800~acidic amino acid	4	SLC25A12, SLC25A13, PSEN1, SLC25A22

transport		
GO:0015849~organic acid transport	8	SLC25A12, CPT1B, CLN3, SLC25A13, PSEN1, SLC25A22, SLC25A1, SLC25A15
GO:0015985~energy coupled proton transport, down electrochemical gradient	6	ATP5D, ATP5F1, ATP5O, ATP5G1, ATP5A1, ATP5G3
GO:0015986~ATP synthesis coupled proton transport	6	ATP5D, ATP5F1, ATP5O, ATP5G1, ATP5A1, ATP5G3
GO:0022900~electron transport chain	12	SLC25A12, NDUFS4, SLC25A13, NDUFB6, NDUFV1, NDUFS8, CYCS, CYC1, NDUFA13, UQCRB, SOD2, GLRX2
GO:0022904~respiratory electron transport chain	8	SLC25A12, NDUFS4, SLC25A13, NDUFB6, NDUFV1, NDUFS8, UQCRB, SOD2
GO:0031667~response to nutrient levels	7	BCKDHA, CLN3, PSEN1, ACADS, BCL2, BCKDHB, SOD2
GO:0032469~endoplasmic reticulum calcium ion homeostasis	4	BAK1, PSEN1, BCL2, BAX
GO:0034440~lipid oxidation	6	CPT1B, ACADS, ECHS1, BDH2, DECR1, CPT1A
GO:0034614~cellular response to reactive oxygen species	3	GPX4, PRDX5, SOD2
GO:0042391~regulation of membrane potential	6	BAK1, CLN3, PSEN1, BCL2, BAX, SOD2
GO:0042594~response to starvation	3	CLN3, PSEN1, ACADS
GO:0042773~ATP synthesis coupled electron transport	5	NDUFS4, NDUFB6, NDUFV1, NDUFS8, UQCRB
GO:0042775~mitochondrial ATP synthesis coupled electron transport	5	NDUFS4, NDUFB6, NDUFV1, NDUFS8, UQCRB
GO:0043436~oxoacid metabolic process	28	BCAT2, PTGES2, NFS1, ECHS1, MTHFD1, GLS2, CKMT2, IDH2, MARS2, ALDH4A1, BDH2, POLG2, SUCLA2, HADH, BCKDHA, CPT1B, CLN3, ACO2, ACADS, SUCLG1, MCAT, CS, BCKDHB, DECR1, PCK2, CPT1A, FPGS, SLC25A15
GO:0044248~cellular catabolic process	28	ATP5D, BCAT2, ECHS1, MTHFD1, LONP1, GPX4, IDH2, ALDH4A1, BDH2, ATP5O, SUCLA2, BCKDHA, CPT1B, CLN3, ACO2, ACADS, SUCLG1, CYCS, CS, BCKDHB, MUL1, DECR1, CPT1A, NNT, PSEN1, BAX, ABAT, ATP5A1
GO:0044260~cellular macromolecule metabolic process	38	TUFM, MRPL42, D2HGDH, GRPEL1, MRPL41, MRPS12, NFS1, MIPEP, HSCB, GLRX2, LONP1, BCL2, TRAK1, MARS2,

		TFB2M, POLG2, DNAJA3, HSPA9, PDK1, CLN3, MRPL51, PHB, PDK4, CYCS, RAF1, MUL1, MRPS21, ESR2, MSRB3, SOD2, MRPL23, MRPS18C, PSEN1, L2HGDH, TSFM, BAX, LRPPRC, DUT
GO:0044262~cellular carbohydrate metabolic process	9	PDK1, PSEN1, PDK4, TRAK1, CS, IDH2, PCK2, OGDH, CPT1A
GO:0044271~nitrogen compound biosynthetic process	15	ATP5D, BCAT2, AK3, NFS1, ATP5F1, ATP5G1, ATP5G3, MTHFD1, SLC25A13, ALDH4A1, ATP5O, ATP5A1, FPGS, SUCLA2, SLC25A15
GO:0045039~protein import into mitochondrial inner membrane	3	TIMM9, TIMM10, TIMM13
GO:0046034~ATP metabolic process	9	ATP5D, LONP1, SLC25A13, ATP5F1, AK3, ATP5O, ATP5G1, ATP5A1, ATP5G3
GO:0046902~regulation of mitochondrial membrane permeability	4	BID, BAK1, BCL2, BAX
GO:0046942~carboxylic acid transport	8	SLC25A12, CPT1B, CLN3, SLC25A13, PSEN1, SLC25A22, SLC25A1, SLC25A15
GO:0048545~response to steroid hormone stimulus	6	BCKDHA, ACADS, GPX4, BCL2, BCKDHB, ESR2
GO:0051171~regulation of nitrogen compound metabolic process	12	BCKDHA, PTGES2, PHB, TRAK1, NDUFA13, TFB2M, ATPIF1, ESR2, DNAJA3, LRPPRC, SOD2, GLRX2
GO:0051345~positive regulation of hydrolase activity	6	BAK1, BBC3, BAX, CYCS, MUL1, DNAJA3
GO:0051881~regulation of mitochondrial membrane potential	4	BAK1, BCL2, BAX, SOD2
GO:0070584~mitochondrion morphogenesis	4	BAK1, FIS1, BAX, MUL1
GO:0070585~protein localization in mitochondrion	9	BID, GRPEL1, TIMM17A, TIMM9, TIMM10, TIMM13, MIPEP, TIMM44, TOMM34

Appendix Table S3. Metabolic parameters in WT and *Tfe3* KO mice fed a HFD for 18 weeks. ALT, AST, ALP, CPK, LDH and glucose values are reported in U/L along with the relative *p* value calculated by Student's two-tailed *t*-test.

	**ALT**	**AST**	**ALP**	**CPK**	**LDH**	**Glucose**
**WT**	318±89.8	259.5±76.6	126±19.5	375.75±76.4	611.25±119.8	284.33±25.5
**KO**	563.3±124.4	686.33±187.6	299.5±64.8	215.5±62.2	3289±1334.7	171±21.2
p value	0.186	0.089	0.042	0.155	0.094	0.0417
**WT HDAd-TFE3 early**	99.75±26.7	127.75±18.9	70.75±18.9	556.5±174.6	373.75±76.8	262.5±23.1
p value	0.058	0.146	0.0443	0.3796	0.146	0.898
**WT HDAd-TFE3 Late**	96±30.3	139.7±27	118.75±17.0	259.25±98.7	365.3±54.8	212.25±21.7
p value	0.100	0.260	0.789	0.387	0.165	0.313

Appendix Table S4. Primers used for qPCR analysis

Gene		Sequences (5'->3')
Tfe3	forward	AGGATCAAAGAGCTGGGCAC
	reverse	CCGGCTCTCCAGGTCTTTG
Pepck	forward	GGCGATGACATTGCCTGGATGA
	reverse	TGTCTTCACTGAGGTGCCAGGA
G6Pc	forward	TGGTAGCCCTGTCTTTCTTTG
	reverse	TTCCAGCATTCACACTTTCCT
Gsk3	forward	TGGCAGCAAGGTAACCACAG
	reverse	CGGTTCTTAAATCGCTTGTCCTG
Opa1	forward	ATACTGGGATCTGCTGTTGG
	reverse	AAGTCAGGCACAATCCACTT
Drp1	forward	TCAGATCGTCGTAGTGGGAA
	reverse	TCTTCTGGTGAAACGTGGAC
Fis1	forward	AAGTATGTGCGAGGGCTGT
	reverse	TGCCTACCAGTCCATCTTTC
Mfn1	forward	CCTACTGCTCCTTCTAACCCA
	reverse	AGGGACGCCAATCCTGTGA
Mfn2	forward	ATGTTACCACGGAGCTGGAC
	reverse	AACTGCTTCTCCGTCTGCAT
Cd36	forward	GGACATTGAGATTCTTTTCCTCTG
	reverse	GCAAAGGCATTGGCTGGAAGAAC
Cpt1a	forward	GGCATAAACGCAGAGCATTCCTG
	reverse	CAGTGTCCATCCTCTGAGTAGC
Cyp7a1	forward	CACCATTCCTGCAACCTTCTGG
	reverse	ATGGCATTCCCTCCAGAGCTGA
Fgf21	forward	ATCAGGGAGGATGGAACAGTGG
	reverse	AGCTCCATCTGGCTGTTGGCAA
Pgc1a	forward	GAATCAAGCCACTACAGACACCG
	reverse	CATCCCTCTTGAGCCTTTCGTG
ApoA4	forward	CAGAAGACGGATGTCACTCAGC
	reverse	AGCTGTACGACAAAGGGCACCA
Cyp17a1	forward	AGCTCTGTGCTGAACTGGATCC
	reverse	AGACGGTGTTCGACTGAAGCCT
Cyp4a10	forward	GCTACTCAAGGCTTTCCAGCAG
	reverse	CCAGAACCATCTAGGAAAGGCAC
Cyp4a14	forward	CAGCTACCAAGGCAGTGTTCAG
	reverse	GGACAAACGTCCATCAGAGGAC
Fasn	forward	AGAAGCCATGTGGGGAAGATT
	reverse	AGCAGGGACAGGACAAGACAA
Srebp1c	forward	AGCTGTCGGGGTAGCGTCTG
	reverse	GAGAGTTGGCACCTGGGCTG
Ucp1	forward	CAAAAACAGAAGGATTGCCGAAA
	reverse	TCTTGGACTGAGTCGTAGAGG

Ucp2	forward	ATGGTTGGTTTCAAGGCCACA
	reverse	TTGGCGGTATCCAGAGGGAA
Ucp3	forward	GTGCTGAGATGGTGACCTACG
	reverse	GCGTTCATGTATCGGGTCTTTA
Ppara	forward	ACCACTACGGAGTTCACGCATG
	reverse	ACCACTACGGAGTTCACGCATG
Pparγ	forward	GTACTGTCGGTTTCAGAAGTGCC
	reverse	ATCTCCGCCAACAGCTTCTCCT
Acot1	forward	ACTACGATGACCTCCCCAAG
	reverse	CATAGCAAGGCCAAGTTCAC
Gapdh	forward	AACACTGAGCATCTCCCTCA
	reverse	GTGGGTGCAGCGAACTTTAT
S16	forward	AGGAGCGATTTGCTGGTGTGG
	reverse	GCTACCAGGGCCTTTGAGATG

Appendix Table S5. Primary antibodies used for immunoblots and staining.

Antigen	Species in which the Ab was raised	Dilution	Source
TFE3	Rabbit	1:1000	Sigma Aldrich
TFEB	Rabbit	1:1000	Bethyl Laboratories
H3	Rabbit	1:3000	Cell Signaling
OXPHOS	Mouse	1:3000	Invitrogen
TOM20	Rabbit	1:250	Santa Cruz
GAPDH	Mouse	1:1000	Santa Cruz
ACTIN	Mouse	1:1000	Novus Biological
DYSTROPHIN	Mouse	1:1000	Novocastra
UCP1	Rabbit	1:5000	Abcam
