# Supplementary material for: TFE3 regulates whole‐body energy metabolism in cooperation with TFEB
Source: EMBO Mol Med. 2017 Mar 10;9(5):605–21. doi: 10.15252/emmm.201607204 (PMC5412821; doi:10.15252/emmm.201607204)
Supplement: Supplementary file 2 — Expanded View Figures PDF [file EMMM-9-605-s002.pdf]

Expanded View Figures

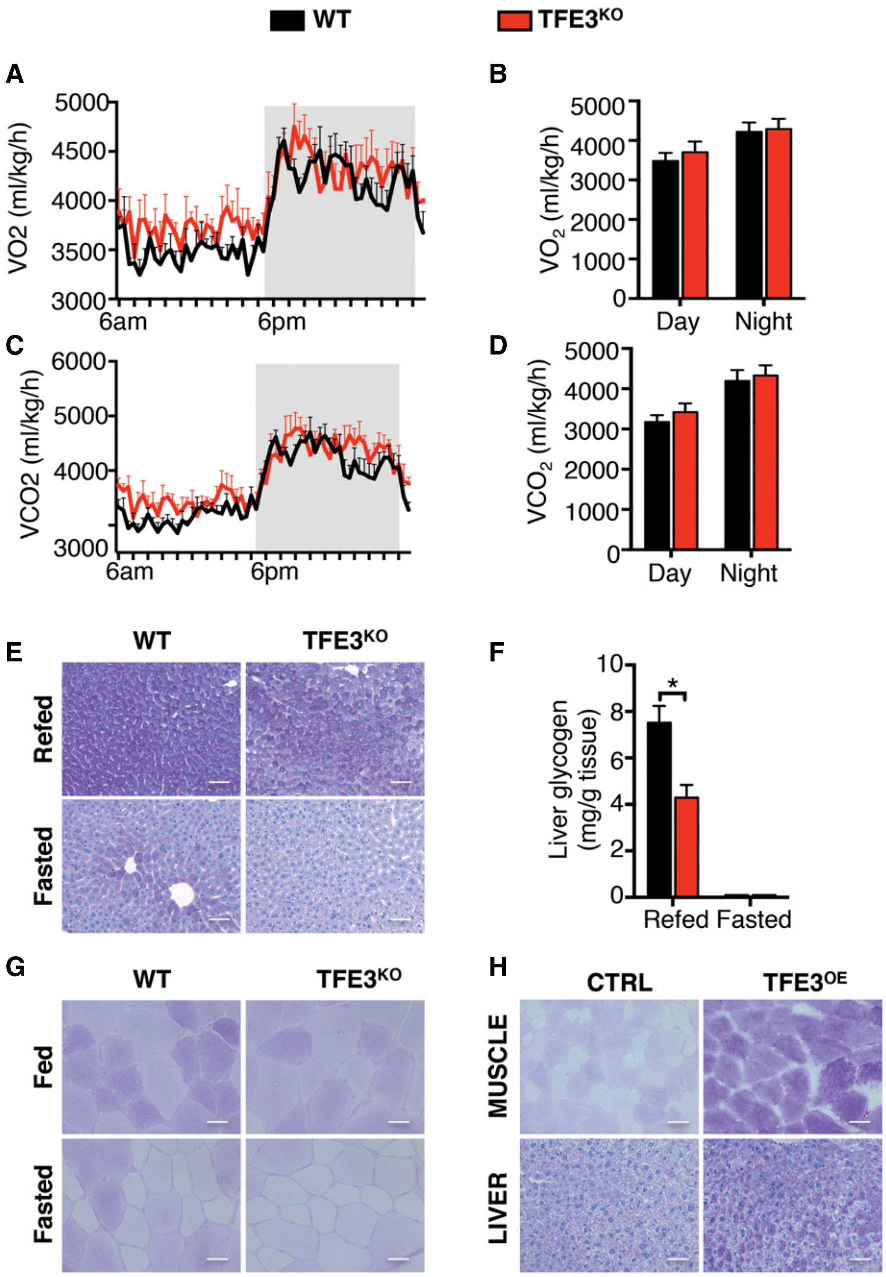

Figure EV1. Metabolic profile of *Tfe3* KO mice.

A Oxygen consumption (V_{O2}) in WT (black line) (*n* = 5) and *Tfe3* KO mice (red line) (*n* = 4) fed a chow diet. Grey areas indicate dark periods (6 PM to 6 AM). Data are presented as mean ± SEM.

B Bar graph represents average V_{O2} values during day and night (*n* = 5 WT and *n* = 4 *Tfe3* KO). Data are presented as mean ± SEM.

C C_{O2} production (V_{CO2}) in WT (black line) (*n* = 5) and *Tfe3* KO mice (red line) (*n* = 4) fed a chow diet. Grey areas indicate dark periods (6 PM to 6 AM). Data are presented as mean ± SEM.

D Bar graph represents average V_{CO2} values during day and night (*n* = 5 WT and *n* = 4 *Tfe3* KO). Data are presented as mean ± SEM.

E, F Periodic acid–Schiff (PAS) staining of liver (E) and relative glycogen content measurement (F) in 24-h-fasted or 24-h-fasted plus 3 h of refeeding WT and *Tfe3* KO mice (*n* = 4 per group) (scale bars: 50 μm). Data are presented as mean ± SEM. Student's two-tailed *t*-test: **P* = 0.0120.

G PAS staining in muscle sections from fed and 24-h-fasted WT and *Tfe3* KO mice (scale bars: 20 μm).

H Representative PAS images from TFE3-overexpressing muscle and liver (scale bars muscle: 20 μm; scale bars liver: 50 μm).

 © 2017 The Authors

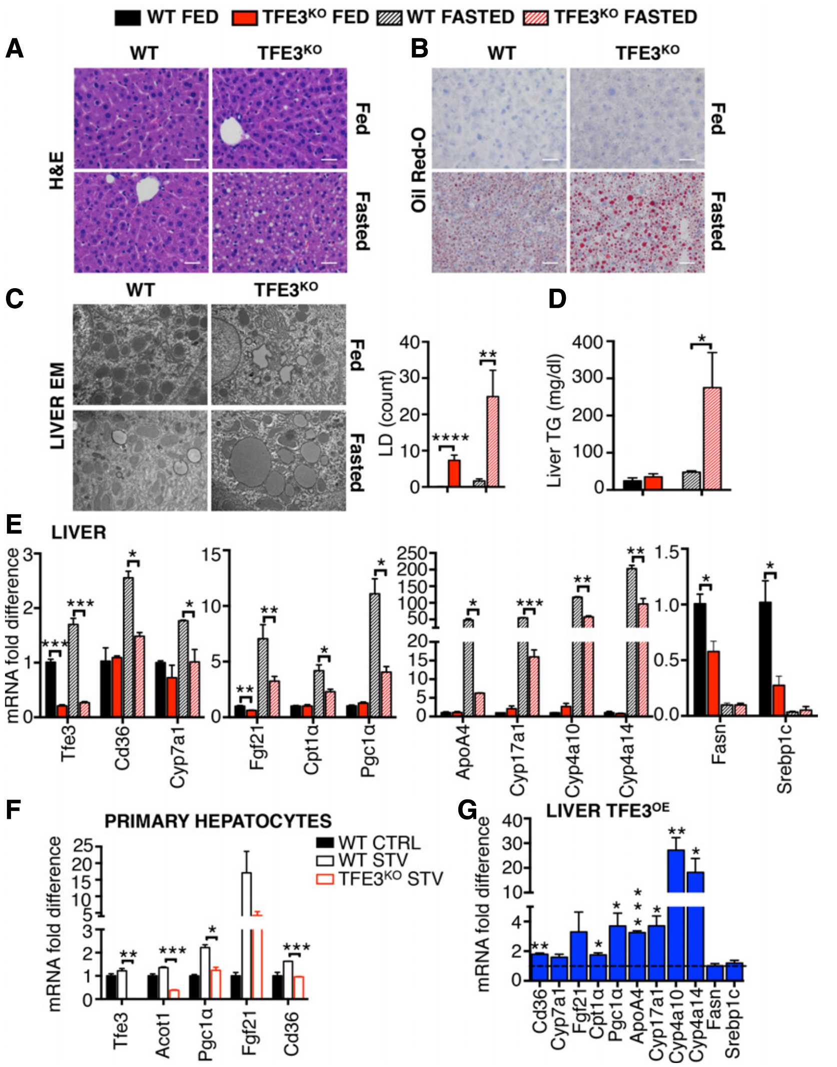

Figure EV2. TFE3 regulates β-oxidation during starvation.

A–D Haematoxylin and eosin (H&E) staining (A), Oil Red O (B) and electron microscopy images (C) with the relative quantification of the lipid droplets and liver triglyceride (TG) levels (D) of livers isolated from fed and 24-h-fasted *Tfe3* KO and control mice ($n = 5$ per group) (scale bars H&E: 20 μm; scale bars Oil Red O: 50 μm). Data are presented as mean ± SEM. Student's two-tailed *t*-test: ****$P < 0.0001$; **$P = 0.0041$; *$P = 0.0288$.

E Quantification of mRNA levels of genes involved in lipid metabolism in livers from WT and *Tfe3* KO mice treated as indicated ($n = 3$ per group). Data are presented as mean ± SEM. Student's two-tailed *t*-test: *Tfe3* ***$P < 0.001$; *Cd36* *$P = 0.0162$; *Cyp7a1* *$P = 0.05$; *Fgf21* **$P = 0.0011$; *Cpt1α* *$P = 0.0307$; *Pgc1α* *$P = 0.0147$; *ApoA4* *$P = 0.0147$; *Cyp17a1* ***$P = 0.0005$; *Cyp4a10* **$P = 0.0056$; *Cyp4a14* **$P = 0.0048$; *Fasn* **$P = 0.0275$; *Srebp1c* *$P = 0.0255$.

F Quantification of mRNA levels of genes involved in lipid metabolism in primary hepatocytes from WT and *Tfe3* KO mice treated as indicated. Data are presented as mean ± SEM. Student's two-tailed *t*-test: *Tfe3* **$P < 0.003$; *Acot1* ***$P = 0.0007$; *Pgc1α* *$P = 0.0159$; *Cd36* ***$P = 0.0002$.

G Expression of genes involved in lipid metabolism in livers from HDAd-PEPCK-*TFE3* injected mice ($n = 3$ per group). Values were normalized to control livers (dashed line). Data are presented as mean ± SEM. Student's two-tailed *t*-test: *Cd36* **$P = 0.0018$; *Cpt1α* *$P = 0.0449$; *Pgc1α* *$P = 0.0207$; *ApoA4* ***$P = 0.0004$; *Cyp17a1* *$P = 0.0162$; *Cyp4a10* **$P = 0.0067$; *Cyp4a14* *$P = 0.0480$.

© 2017 The Authors

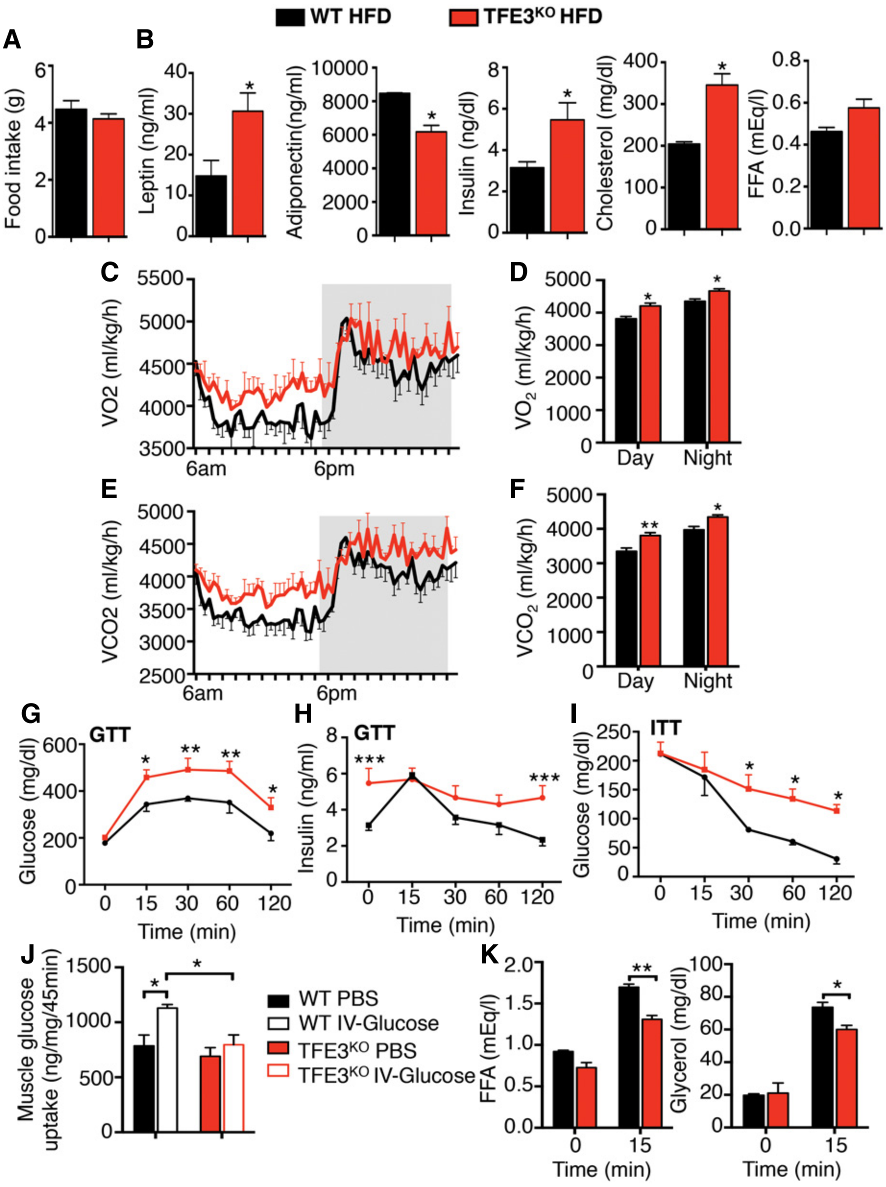

Figure EV3. Metabolic profile of *Tfe3* KO mice fed a HFD.

A, B Food intake (A) (n = 5) and serum panel (B) from WT and *Tfe3* KO mice after 1 month of HFD. Data are presented as mean ± SEM. Student's two-tailed t-test: leptin (n = 3) *P = 0.0451; adiponectin (n = 3) *P = 0.0192; insulin (n = 5) *P = 0.0287; cholesterol (n = 3) *P = 0.0277.

C Oxygen consumption (V_{O2}) in WT (black line) (n = 5) and *Tfe3* KO mice (red line) (n = 5) after 1 month of HFD. Grey areas indicate dark periods (6 PM to 6 AM). Data are presented as mean ± SEM.

D Bar graph represents average V_{O2} values during day and night (n = 5 per group). Data are presented as mean ± SEM. Student's two-tailed t-test: day *P = 0.0122; night *P = 0.0174.

E CO_2 production (V_{CO2}) in WT (black line) (n = 5) and *Tfe3* KO mice (red line) (n = 5) after 1 month of HFD. Grey areas indicate dark periods (6 PM to 6 AM). Data are presented as mean ± SEM.

F Bar graph represents average V_{CO2} values during day and night (n = 5 per group). Data are presented as mean ± SEM. Student's two-tailed t-test: day **P = 0.0076; night *P = 0.0113.

G, H Glucose (G) and insulin (H) levels at the indicated time point after glucose challenge (n = 5 per group). Data are presented as mean ± SEM. ANOVA test followed by *post hoc* Bonferroni test: GTT *P = 0.0106 (15 min), **P = 0.0066 (30 min), **P = 0.0029 (60 min), *P = 0.0144 (120 min); insulin during GTT ***P = 0.0002.

I Glucose levels at the indicated time point after insulin challenge (n = 5 per group). Data are presented as mean ± SEM. ANOVA test followed by *post hoc* Bonferroni test: *P = 0.04 (30 min); *P = 0.03 (60 min); *P = 0.014 (120 min).

J Muscle glucose uptake in control and *Tfe3* KO mice after 1 month of HFD (n = 3 per group). Data are presented as mean ± SEM. Student's two-tailed t-test: WT PBS versus WT IV glucose *P = 0.0294; WT IV glucose versus *Tfe3* KO IV glucose *P = 0.0245.

K *In vivo* lipolysis measured in WT and *Tfe3* KO mice fed a HFD for one month as indicated in the Materials and Methods section (n = 3 per group). Data are presented as mean ± SEM. Student's two-tailed t-test: FFA **P = 0.040; glycerol *P = 0.0417.

 © 2017 The Authors

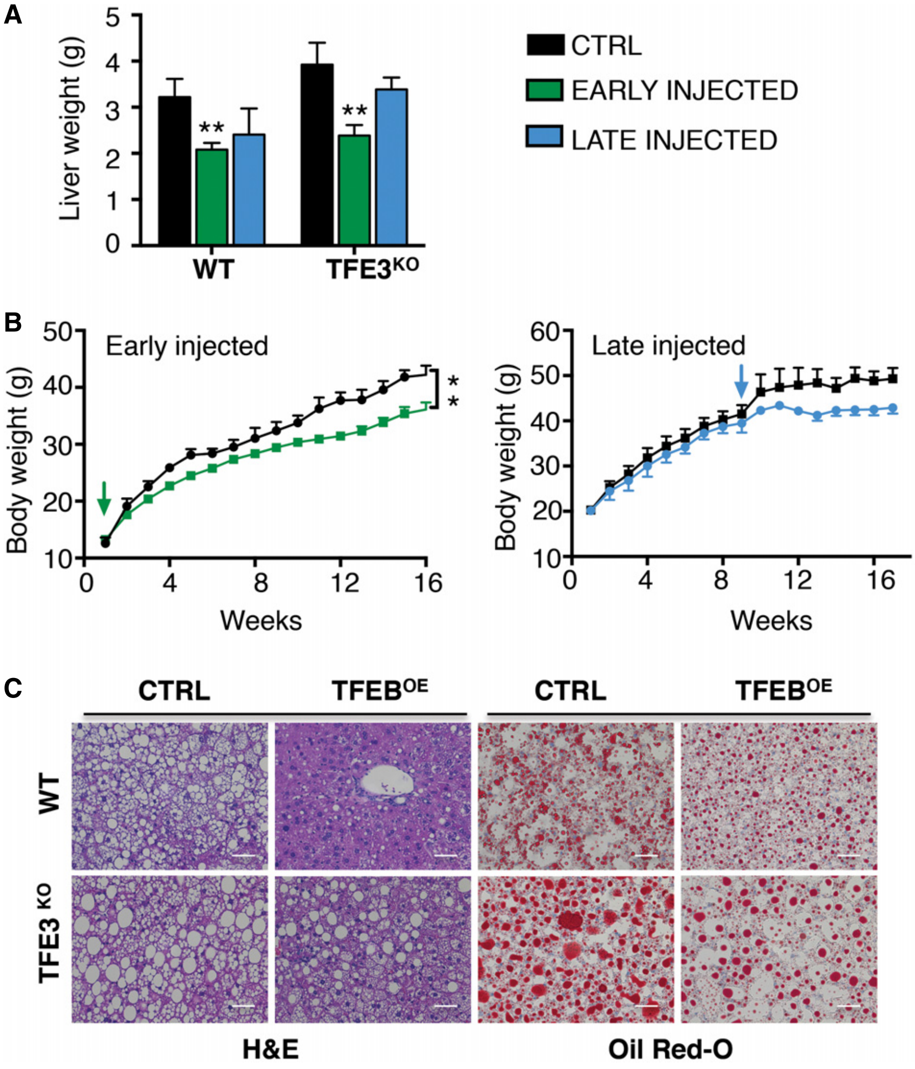

Figure EV4. TFEB overexpression rescues diet-induced obesity in *Tfe3* KO mice.

A Liver weight from WT and *Tfe3* KO mice injected with the HDAd-PEPCK-h*TFEB* prior to HFD administration (early) (n = 13) or 8 weeks into HFD (late) (n = 4) and controls (n = 6) of the indicated genotypes. Data are presented as mean ± SEM. ANOVA test followed by *post hoc* Bonferroni test: WT $^{**}P$ = 0.0032; *Tfe3* KO $^{**}P$ = 0.0041.

B Body weight in *Tfe3* KO mice injected with an HDAd-PEPCK-*TFEB*. Arrows indicate the time of injection. Left panel: controls (n = 4) and early-injected mice (n = 13). Right panel: controls (n = 4) and late-injected mice (n = 3). Data are presented as mean ± SEM. ANOVA test followed by *post hoc* Bonferroni test: early $^{**}P < 0.01$.

C H&E (left panel) and Oil Red O staining (right panel) of liver sections from late-injected mice of the indicated genotypes after 15 weeks of HFD (scale bars: 50 μm).
