# Supplementary figures and images for: TFE3 regulates whole‐body energy metabolism in cooperation with TFEB
Source: EMBO Mol Med. 2017 Mar 10;9(5):605–21. doi: 10.15252/emmm.201607204 (PMC5412821; doi:10.15252/emmm.201607204)

Appendix

Appendix S1B

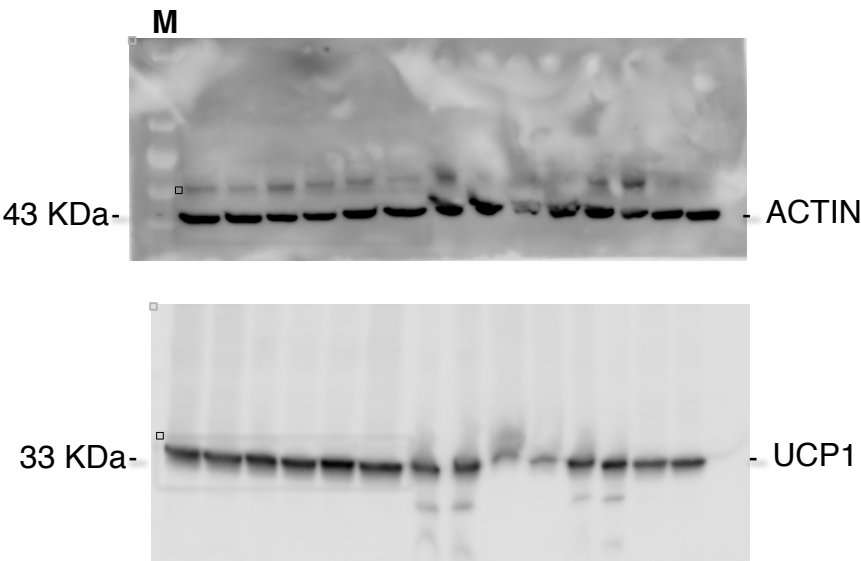

Appendix S1A

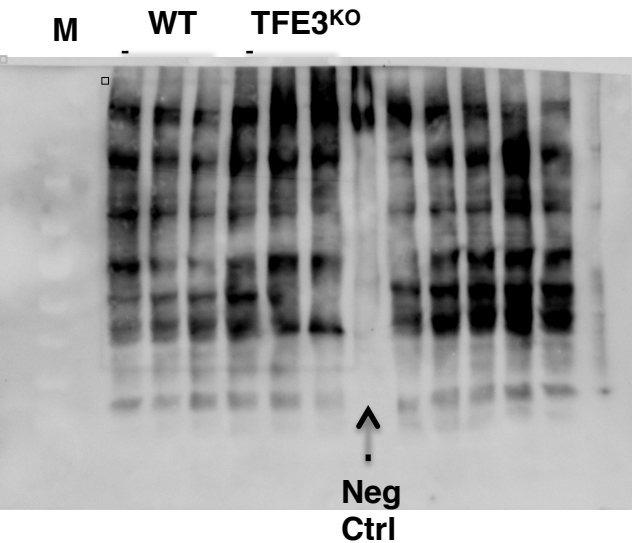

Supplement: Supplementary file 3 — Source Data for Appendix [file EMMM-9-605-s003.zip › EMM_07204_Appendix_source_data.pdf]

Figure 4

Figure 4C

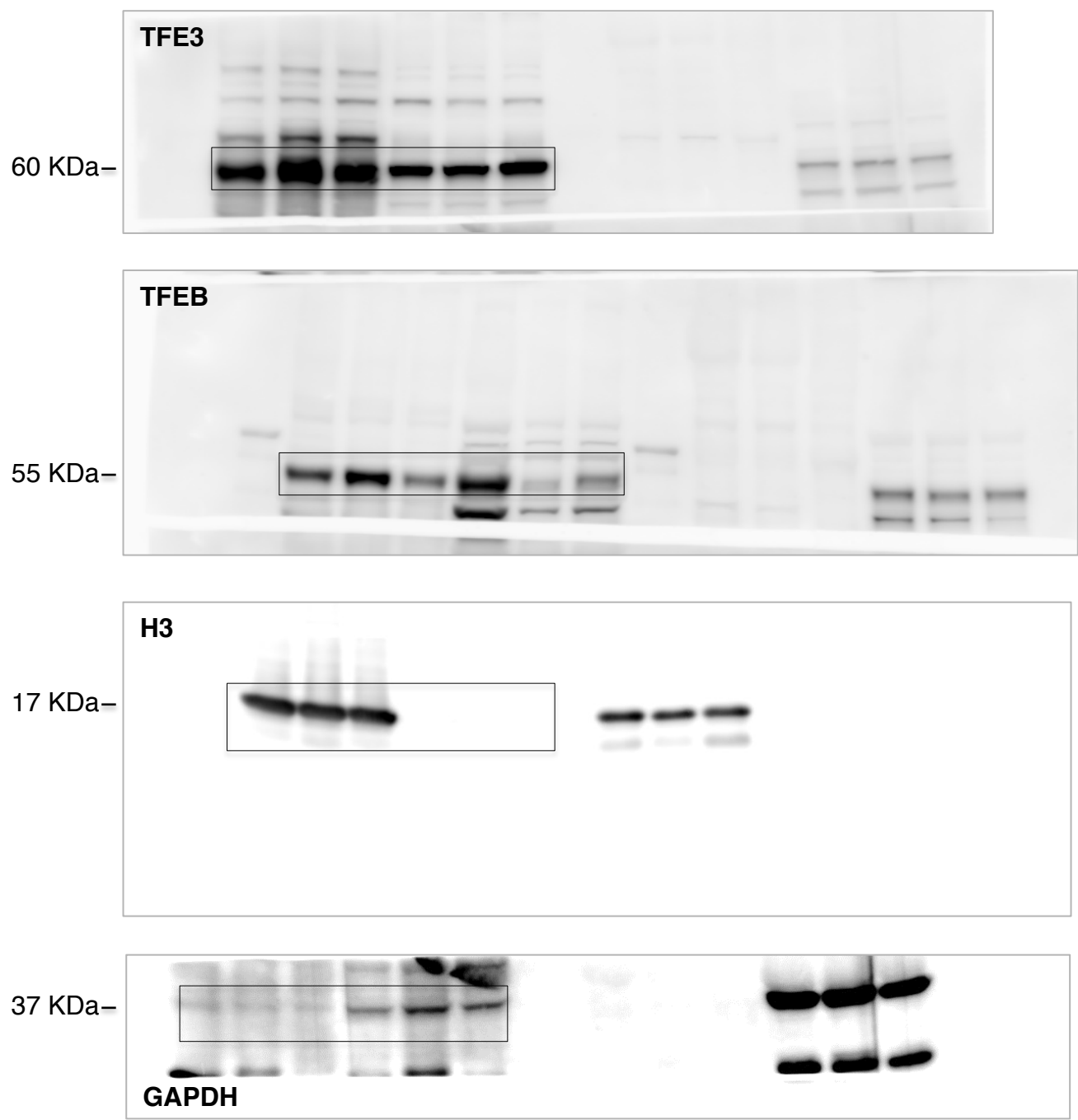

Figure 4

Figure 4D

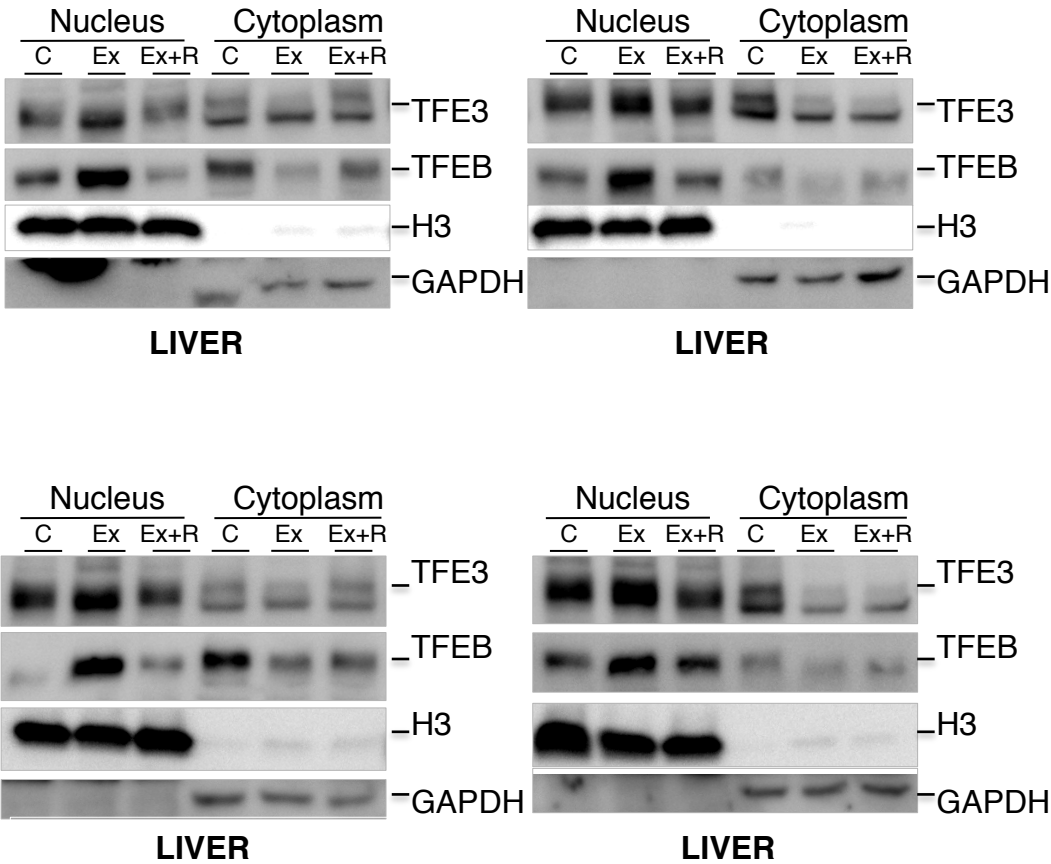

Supplement: Supplementary file 5 — Source Data for Figure 4 [file EMMM-9-605-s004.pdf]

Figure 6

Figure 6A

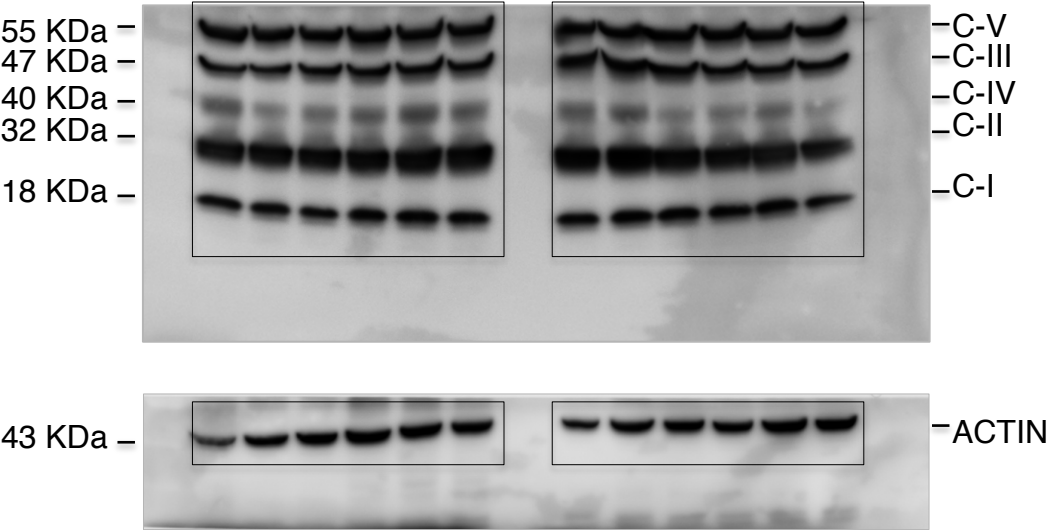

Supplement: Supplementary file 6 — Source Data for Figure 6 [file EMMM-9-605-s005.pdf]
